# Motivational Influences on Performance Monitoring and Cognitive Control Across the Adult Lifespan

**DOI:** 10.3389/fpsyg.2018.01018

**Published:** 2018-06-26

**Authors:** Nicola K. Ferdinand, Daniela Czernochowski

**Affiliations:** ^1^Department of Psychology, Saarland University, Saarbrücken, Germany; ^2^Center for Cognitive Science, Technische Universität Kaiserslautern, Kaiserslautern, Germany

**Keywords:** cognitive control, performance monitoring, adult lifespan, incentives, motivation

## Abstract

Cognitive control refers to the ability to regulate cognitive processing according to the tasks at hand, especially when these are demanding. It includes maintaining and updating relevant information in working memory, inhibiting irrelevant information, and flexibly switching between tasks. Performance monitoring denotes the processing of feedback from the environment and the detection of errors or other unexpected events and signals when cognitive control needs to be exerted. These two aspects of behavioral adaptation critically rely on the integrity of the frontal lobes, which are known to show pronounced age-related performance decrements. By contrast, there is evidence that processing of rewards remains relatively intact across the adult lifespan. Hence, motivation may play an important role in modulating or even counteracting age-related changes in cognitive control functions. To answer this question, neuroscientific data can be particularly useful to uncover potential underlying mechanisms beyond behavioral outcome. The aims of this article are twofold: First, to review and systematize the extant literature on how motivational incentives can modulate performance monitoring and cognitive control in young and older adults. Second, to demonstrate that important pieces of empirical data are currently missing for the evaluation of this central question, specifically in old age. Hence, we would like to stimulate further research uncovering potential mechanisms underlying motivation-cognition interactions in young and in particular in older adults and investigating whether or not those can help to ameliorate age-related impairments.

## Introduction

It is well known that many cognitive functions, mainly those from the domain of fluid intelligence, decline with increasing age (Baltes, 1997; Baltes et al., 1999). By contrast, there is evidence that affective and motivational information processing remains relatively intact across the adult lifespan. Neuroscientific research has corroborated these findings by revealing diverging trajectories of cognitive and affective neural substrates, with cognitive prefrontal circuits being more strongly affected by aging than affective ones (for reviews, see Eppinger et al., 2011; Mata et al., 2011; Mather, 2012). Recent research has also demonstrated that motivational influences can modulate cognitive functioning and enhance performance in diverse cognitive tasks (for a review, see Braver et al., 2014), for instance that rewards can improve memory performance (Wittmann et al., 2005; Adcock et al., 2006; Halsband et al., 2012). This interaction of motivation and cognition is particularly relevant for aging research as it may provide an opportunity to ameliorate age-related impairments.

The goal of this article is to review and systematize recent research examining the influence of motivational incentives on performance monitoring and cognitive control across the adult lifespan. Performance monitoring denotes the processing of environmental feedback, the detection and processing of errors and of unexpected events. It is an important prerequisite for the flexible adaptation of behavior to varying situational demands because it signals when our behavior was inadequate or did not lead to the expected goal. It is also a precondition for the implementation of cognitive control because it signals when (more) cognitive control is needed. Cognitive control is the ability to guide one’s own behavior and cognitive processes in a goal-directed way. It is a multidimensional construct comprising several core components, including the ability to select relevant information, to keep it active in working memory and protect it against irrelevant information (Miyake et al., 2000; Hofmann et al., 2012; Miyake and Friedman, 2012; Grange and Houghton, 2014).

These fundamental cognitive functions constitute the basis for more complex abilities like adaptive behavior, rational decision making, or self-regulation (Hofmann et al., 2012). They also rely heavily on prefrontal cortex (PFC) functioning and are highly susceptible to age-related decline (e.g., West, 1996; Braver and Barch, 2002; Miller and Cohen, 2001; Paxton et al., 2008). Hence, it is an important question whether motivation can modulate age-related decline in these abilities. In this article, we will first address the question of how the processing of motivational incentives changes across the adult lifespan (see section “Processing of Rewards”). We will then review the literature on motivational influences on performance monitoring (see section “Motivational Influences on Performance Monitoring”) and cognitive control (see section “Motivational Influences on Cognitive Control”). These sections show that age-related differences of motivational impact on these two aspects of cognition are scarcely examined up-to-date (for an overview, see **Tables 1, 2**). Thus, we will briefly summarize research findings in young adults and focus on older participants whenever possible. Finally, we will discuss the extant empirical findings on motivation–cognition interactions in the light of aging models on cognitive control functioning (see section “Potential Mechanisms of Motivation on Performance Monitoring and Cognitive Control in Old Age”) and discuss caveats and limitations that this line of research is confronted with, in particular the lack of systematic evaluations of cognition-motivation interactions in older adults (see section “Open Issues, Caveats, and Future Directions”).

## Processing of Rewards

A fundamental question is whether the processing of rewards and punishments changes across the adult lifespan. If younger and older adults process these incentives differently, this fundamental difference may have important implications for how and whether incentives can exert their influence over other cognitive processes.

From a theoretical perspective, there is reason to believe that rewards and punishments change their motivational value over the lifespan. For instance, the socio-emotional selectivity theory (Mather and Carstensen, 2005; Reed and Carstensen, 2012) assumes that there is an emphasis on emotional satisfaction and well-being in old age when future time horizons are restricted and that this is the reason for an age-related positivity shift, i.e., the preferred processing of positive information. This effect has been found mainly in the domains of attention (Mather and Carstensen, 2005; Isaacowitz et al., 2006) and memory (Kennedy et al., 2004; Grady et al., 2007). For instance, when shown positive, negative, and neutral pictures, older adults recall more positive pictures and fewer negative pictures than younger adults (Charles et al., 2003). However, it remains unclear to what extent the positivity-effect generalizes to other domains of cognitive functioning. The same argument can be put forward concerning the model of selection, optimization and compensation by Baltes and colleagues (e.g., Baltes and Baltes, 1990; Ebner et al., 2006). Similar to the socio-emotional selectivity theory, this model assumes that positive and negative motivational information differentially affect cognition in older adults. However, it proposes that the prevention of losses is more relevant to older adults than the receipt of gains (for a similar view, see Brandtstädter, 2009). Because age-related differences in reward processing *per se* are not the main focus of this article, in the following we will present only a short summary on the main findings of this research area and direct the interested reader to the respective literature (for reviews, see Eppinger et al., 2011; Mata et al., 2011).

### Reward Anticipation and Delivery

Existing functional magnetic resonance imaging (fMRI) studies have examined the activation of reward networks during the anticipation and the delivery of rewards by means of incentive delay tasks. In young adults, these studies have consistently found that anticipation and delivery of rewards and punishments activate parts of the ventral striatum (bilateral caudate nucleus and bilateral putamen), the insula, the dorsal midbrain, and the orbitofrontal cortex (e.g., Delgado et al., 2000, 2003; Knutson et al., 2000; Breiter et al., 2001; Nieuwenhuis et al., 2005; Elliott et al., 2008; Rolls and Grabenhorst, 2008; for a review, see Delgado, 2007). This line of research has also shown that reward-network responses during anticipation and outcome processing show relatively little age-related change. For example, Samanez-Larkin et al. (2007) examined anticipation of gains and losses in a monetary incentive delay task. They found that older adults demonstrated preserved gain anticipation in the ventral striatum and the insula (for similar results, see Rademacher et al., 2014; Spaniol et al., 2015), but also a reduced activation in the nucleus caudatus and the insula during loss anticipation as compared to younger adults. This latter finding was also consistent with older adults’ self-report indicating that they experienced reduced negative affect (Samanez-Larkin et al., 2007). Cox et al. (2008) focused on the delivery of monetary rewards and punishments in a card-guessing task. They found that older adults showed the same activation foci and temporal dynamics during reward and punishment delivery as younger adults. Interestingly, both studies hint at the possibility of a positivity bias in old age, consistent with the socio-emotional selectivity theory (Mather and Carstensen, 2005; Reed and Carstensen, 2012). While Samanez-Larkin et al. (2007) found that older adults may be less sensitive to loss cues than younger adults, Cox et al. (2008) found a trend for older adults to show a decreased response to punishments. However, more research is needed to corroborate these rather subtle effects.

### Reward Prediction Errors

In contrast to reward processing *per se*, older adults show decreased functioning in processing reward prediction errors, i.e., the difference between expected and actual rewards (for a review, see Mata et al., 2011). This is most evident during reinforcement learning, where learning is induced by rewards or punishments that indicate whether an event has been better or worse than predicted. For instance, Eppinger et al. (2013) examined younger and older participants in a reinforcement learning task with a reward (win vs. no-win) and a punishment (loss vs. no-loss) condition using fMRI. They found that older adults showed less learning in combination with reduced activity in the ventromedial PFC in the reward condition but not in the punishment condition. Moreover, they found a reduced sensitivity to reward prediction errors in the ventral striatum in older adults (Eppinger et al., 2013; for similar results, see Schott et al., 2007; Mell et al., 2009; Samanez-Larkin et al., 2014).

Reward prediction errors have also been examined by means of event-related potentials (ERPs) and several components have been associated with their detection (for reviews, see Folstein and Van Petten, 2008; Gehring et al., 2012). Most important in the present context is the feedback-related negativity (FRN), which is thought to originate (at least in part) from the anterior cingulate cortex (Miltner et al., 1997; Ferdinand and Opitz, 2014). It is usually measured over fronto-central brain areas after participants receive unexpected feedback or rewards/punishments (e.g., Gehring and Willoughby, 2002; Holroyd and Coles, 2002; Ferdinand et al., 2012). In line with the above fMRI findings, ERP studies have demonstrated that older adults generally show reduced feedback negativities (e.g., Mathalon et al., 2003; Mathewson et al., 2005; Hämmerer et al., 2010; Bellebaum et al., 2011) as compared to younger adults. According to Nieuwenhuis et al. (2002), this is the result of a weakened reinforcement learning signal from the dopamine system to the mediofrontal cortex, specifically the anterior cingulate.

As a consequence of the above findings, it has been suggested that the sensitivity to rewards and previously learned reward associations remain intact over the adult lifespan, whereas a network of neural systems that supports novel reward learning changes with age (e.g., Samanez-Larkin et al., 2014). Specifically, an age-related reduction in structural connectivity between the striatum and the PFC has been found (Samanez-Larkin et al., 2012). This reduction in connectivity may influence the dynamic updating of reward predictions and thus explain the age-related impairments in reward prediction error processing (Eppinger et al., 2011). This line of argumentation is also corroborated by ERP studies showing that age differences in the FRN are reduced when the task’s difficulty level is adaptive (e.g., Eppinger et al., 2008; Ferdinand and Kray, 2013). This implies that neither reward processing nor prediction error processing *per se* is impaired in old age, but that the decreased availability of processing resources leads to the observed age effects in reward prediction error processing.

## Motivational Influences on Performance Monitoring

Performance monitoring includes the detection and processing of errors and external feedback as well as the detection of other unexpected events in our environment. It is a crucial prerequisite to flexibly adjust our behavior to different situational demands, because it signals when behavior has not resulted in the desired outcome. By this, it is an important marker indicating that heightened cognitive control is necessary. There are several theories about how performance monitoring contributes to behavioral changes (for an overview, see Alexander and Brown, 2010). What is common to most of them is that we make predictions about the outcome of events and compare them to the actual outcome. When this comparison results in a mismatch, the brain generates a (reward) prediction error signal, which is then used to adjust our behavior and update our expectancies for the future. Hence, a core component of performance monitoring is the generation of a (reward) prediction error.

Performance monitoring involves a cognitive as well as an affective component, which are probably inseparably interwoven in daily life. For instance, receiving negative feedback from another person regarding our behavior is rarely just purely informative, but also has an emotional impact. Therefore, a separation of performance monitoring and motivational influences on performance monitoring is difficult. This is also reflected in recent research where oftentimes no differentiation is made between “abstract performance feedback” and “reward or punishment feedback.” Instead, it is assumed that even abstract performance feedback has a rewarding or punishing effect and thus has a motivational impact. Conversely, it is oftentimes implicitly assumed that monetary gains can be used as positive feedback and losses as negative feedback without affecting the basic monitoring processes (cf. Holroyd and Coles, 2002). For this reason, the effect of motivational influences on error and feedback processing has typically been examined by manipulating the amount of the reward that can be won or lost. Other manipulations include comparing different types of rewarding feedback and inducing a motivational mindset by presenting a win or loss cue prior to the task at hand. These three types of motivational manipulations will be reviewed in the following (see **Table 1**).

**Table 1:** Behavioral and neuroscientific studies examining age differences in performance monitoring.

Study	Method	Age groups	Motivational influence	Paradigm	Main results
Gorlick et al., 2013	Behavioral	18–35 and 60–82 years	Cognitive feedback (point gain vs. point loss) vs. social feedback (happy vs. angry faces)	Rule learning and set shifting via feedback	Experiment 1 (face feedback): • Minimal load on cognitive control: happy-face feedback attenuated age-related deficits in initial rule learning and angry-face feedback led to age-related deficits in initial rule learning and set shifting
					• High load on cognitive control: angry-face feedback attenuated age-related deficits in initial rule learning and set shifting whereas happy-face feedback led to age-related deficits in initial rule learning and set shifting
					Experiment 2 (point feedback): • Age-related deficits in initial rule learning and set shifting under low and high cognitive load for point-gain and point-loss conditions
Kardos et al., 2016a	ERPs	21–28 and 62–72 years	Points accumulated during experiment are added to participation fee	Balloon Analog Risk Task	• In young, reward positivity increased as function of reward contingencies with largest amplitude for rewarding feedback followed by the decision to stop
					• Older adults characterized by hesitation and more deliberative decision making, reward positivity did not reflect the effect of reward structure
Kardos et al., 2016b	ERPs	18–32 and	Two amounts of monetary	Gambling task	• Riskier choices after negative feedback
		62–72 years	gains/losses		• In young adults, FRN was indicator of goodness of outcome (loss or gain), P3 showed a complex picture of feedback evaluation with selective sensitivity to large amount of gains
					• In older adults, outcome valence had no effect on FRN, P3 was insensitive of the complex outcome properties
Nashiro et al., 2011	Behavioral	Exp 1: 18–25 and 62–83 years Exp 2: 18–24 and 69–93 years Exp 3: 18–26 years	Angry and happy faces as feedback vs. more or less points	Learning and set shifting task	• Older adults made more errors than younger adults in the angry face feedback condition, but no age differences in happy face feedback condition

### Reward Magnitude

In fMRI studies, evidence has accumulated showing that activity in the striatum varies as a linear function of reward magnitude. For example, Bjork et al. (2010) found that the ventral striatum was sensitive to the amount of monetary gains in young adults during the anticipation and the receipt of gains and losses in a monetary incentive delay task (see also Bjork et al., 2004; Izuma et al., 2008). Similarly, Delgado et al. (2003) found that in young participants the dorsal striatum, more specifically the nucleus caudate in the left hemisphere of the brain, was sensitive to both magnitude and valence: the highest activations were found for high rewards, followed by small rewards. Small punishments elicited even less activation of the nucleus caudate and the lowest activations were associated with large punishments.

In studies using ERPs, however, the results are far less consistent. Some studies found that the fast detection process as indexed by the FRN is not modulated by the size of gains or losses in young adults (Holroyd et al., 2004; Sato et al., 2005). Others found that the FRN in response to negative feedback was larger the larger the amount of money that could have been gained (Bellebaum et al., 2010) or that it was only sensitive to the magnitude of wins, but not losses (Zottoli and Grose-Fifer, 2012; Lole et al., 2013; Grose-Fifer et al., 2014). One explanation for these mixed findings could be related to the gender of the participants: the effects of reward magnitude on FRN in mixed-gender samples might be driven primarily by males, because two of the above studies found a larger FRN to small as compared to large wins for young adult males only (Zottoli and Grose-Fifer, 2012; Grose-Fifer et al., 2014). This might indicate that large wins are especially salient for young adult males, a sample that is known to be highly risk seeking and reward driven. Another explanation for the inconsistent findings might be due to the fact that most of the above studies that did not find a sensitivity of the FRN to reward magnitude did not control for subjects’ expectancies. However, expectancies are of critical importance because a prediction error is the deviation of an actual outcome from an expected one (cf. Bellebaum et al., 2010). In line with this view, Hajcak et al. (2007) found a gradual increase in FRN amplitude with increasing prediction error when participants’ expectancies were taken into account. Taken together, this implies that the FRN is not sensitive to reward magnitude *per se*, but to the size of the prediction error. This might or might not coincide with reward magnitude, depending on the study design. In contrast to the FRN, reward magnitude has been found to influence a later ERP component, the P300. The P300 is a large positive deflection following the FRN that is probably associated with working memory updating after unexpected task-relevant events (e.g., Polich, 2007) and which reflects a slower and more elaborate feedback evaluation process than the FRN (e.g., Ferdinand and Kray, 2013). Sato et al. (2005) found that P300 amplitude increased with reward magnitude, irrespective of valence (for a similar result, see Grose-Fifer et al., 2014; Kardos et al., 2016b).

Studies examining this question in older adults are scarce. Kardos et al. (2016b) examined younger and older adults in a two-choice gambling task with two amounts of monetary stakes. Their results in the sample of young adults closely resemble those by Sato et al. (2005) reported above, by demonstrating that the FRN reflected the goodness of the outcome in a binary fashion (loss vs. gain), while the P3 showed a complex picture of feedback evaluation with selective sensitivity to large gains. In contrast, in older adults outcome valence had no effect on FRN amplitude and the P300 was insensitive to outcome magnitude. Thus, it may be the case that due to limited processing resources, older adults strategically focus on the most important aspects of a feedback stimulus (cf. Ferdinand and Kray, 2013). Thus, adding more information to the feedback stimulus (magnitude information in addition to valence information) may not be the optimal way to vary reward magnitude in older adults (see also Herbert et al., 2011). In a similar vein, Kardos et al. (2016a) examined risk taking behavior in younger and older adults using the Balloon Analog Risk Task in which each pump on a virtual balloon increased the probability of a balloon burst but also increased the chance to earn a larger reward. Again, the positivity after reward feedback increased as a function of reward contingencies with the largest positivity after reward feedback followed by the decision to stop inflating the balloon. This graded ERP response was not found in older adults. Also, older adults showed more hesitation and more deliberative decision making. Thus, the lack of differentiation in the ERP response to rewarding feedback might be related to more uncertainty and variability in decision making under risky circumstances.

Taken together, there is clear evidence from neuroscientific studies that younger adults process reward magnitude. It also modulates performance monitoring, although it is not yet clear which specific monitoring mechanisms are affected most. The few cross-sectional studies show a markedly different result pattern for older adults which seem to be insensitive to reward magnitude.

### Incentive Cues

A second possibility for examining motivation-cognition interactions in performance monitoring is to present a win or loss cue prior to the task at hand and thus induce a motivational mindset. This might be the most promising approach to examine the effect of incentives on performance monitoring because the same cognitive task can be examined in distinct motivational conditions (gain or loss mindset) and compared to a neutral condition without incentives. Hajcak et al. (2005b), for instance, examined error monitoring in a flanker task in young adults. Each trial was preceded by a cue indicating the value of the trial (5 or 100 points that were converted to money at the end of the experiment). While performance did not differ between the two trial types, the size of the error negativity (ERN/Ne; Falkenstein et al., 1990; Gehring et al., 1993), an ERP component signaling the detection of a committed error and the need for behavioral adaptation, was substantially larger in high-value trials (Hajcak et al., 2005a). Pailing and Segalowitz (2004) examined error monitoring in a letter discrimination task with young adults and compared conditions in which potential rewards could be gained with a no reward condition. In contrast to Hajcak et al. (2005a), they found that monetary incentives had a motivational effect on behavior and led to better task performance, although this was not reflected in a modulation of the ERN. However, the authors assumed that this could be due to a ceiling effect because their participants were highly motivated in all study conditions. To reconcile these seemingly contradictory findings, one could speculate that the two studies above differed in terms of task difficulty. Motivational incentives presumably can only impact performance when there is sufficient room for improvement. When participants are already performing at floor or at ceiling because either the task is too hard or too easy, incentives might still have an effect on the salience of an error as reflected in the ERN but not on performance (see also section “Open Issues, Caveats, and Future Directions”). As for feedback processing, Threadgill and Gable (2016) presented cues indicating a potential monetary reward or a neutral cue at the start of each trial in a flanker task. They found that in young adults, a reward cue in comparison to a neutral cue sped up performance and led to a larger reward positivity, an ERP component in the time window of the FRN reflecting processing of positive outcomes. Similarly, Flores et al. (2015) compared feedback processing in a monetary and a social incentive delay task and found a larger FRN and P300 in trials with potential rewards (monetary and social) as compared with no potential rewards (see also section “Different types of rewards”).

Taken together, cues signaling potential rewards seem to be effective motivational incentives for young adults and enhance error and feedback processing when there is room to improve performance. To our knowledge, there are no similar studies examining the effect of motivational incentive cues on performance monitoring in old age. However, because performance monitoring is a prerequisite for behavioral adaptation which is impaired in older adults (see section “Reward Prediction Errors”), it would be crucial to know how it could be supported or even improved.

### Different Types of Rewards

Another approach to investigate motivational influences on performance monitoring is to compare the effects of different types of reward feedback. In the context of performance monitoring, the most commonly used types of feedback are abstract performance feedback, e.g., symbols informing participants whether their response has been correct/incorrect, and monetary feedback, i.e., amounts of money won/lost. Other types of feedback are scarcely investigated. However, different types of rewards can have a different subjective value and therefore have different motivational impact. Which reward is perceived as most motivating could also change across the lifespan. For instance, preserved cognitive functioning plays a key role in old age (Baltes and Baltes, 1990; Brandtstädter, 2009). Hence, one could speculate that performance feedback, especially in a social context, might be more effective in older adults than monetary rewards, and also one prominent reason for older adults to participate in experimental studies (see also section “Open Issues, Caveats, and Future Directions”).

In line with this idea, some studies have used social stimuli as feedback, like social acceptance or rejection (Davey et al., 2011; Kujawa et al., 2014) or emotional faces (Zhang et al., 2012; Vrticka et al., 2014). In rare cases, primary reinforcers like candy or soft drinks have been used (Luking and Barch, 2013). However, these studies did not include a direct comparison between different types of rewarding feedback, so a potential additional motivational influence of reward feedback as compared to performance feedback cannot be investigated.

Only a handful of studies have explicitly contrasted the effect of different types of rewarding feedback in the same paradigm. Hurlemann et al. (2010) compared abstract performance feedback (green vs. red lights) with social feedback (smiling vs. angry faces) in a behavioral item-category learning paradigm with young adult males in their twenties. They found better performance in conditions with social feedback. Gorlick et al. (2013) examined younger and older adults in a rule learning task with cognitive (point-gain vs. point-loss) and social (happy vs. angry faces) feedback. They did not find performance differences in the two conditions in younger adults. However, although older adults showed age-related impairments in learning, those were substantially smaller in the condition with social feedback. This effect showed complex interactions with feedback valence (positive vs. negative) and working memory demands: in conditions of low working memory demands, age-effects were reduced by positive but enlarged by negative social feedback. Thus, while older adults profited from positive social feedback, negative social feedback had deteriorating effects. For high working memory load, the opposite pattern emerged. Notably, this effect was not found for cognitive feedback, emphasizing a special role of social feedback in old age (see also Nashiro et al., 2011).

Importantly, although these behavioral studies use paradigms that are commonly used in the area of performance monitoring, one can only speculate which specific cognitive processes are affected by the incentives in these tasks. Here, neuroscientific measures can be extremely helpful. To this effect, Hajcak et al. (2005b) used ERPs to examine error monitoring in a flanker task with university students in a social evaluation and a control condition. Although no performance difference was found between the two conditions, in the social evaluation condition the ERN after committed errors was substantially larger, i.e., the monitoring system was much more sensitive. As for feedback processing, Dekkers et al. (2015) conducted a social-judgment and an age-judgment task with young women. In the social-judgment task, participants had to judge whether they expected a person to like them or not. In the age-judgment task they were to judge whether the person was their age or not. Afterwards they received feedback about their judgment (yes/no). While there was no effect of task condition on the FRN, a greater P3 was found in the social-judgment compared with the age-judgment task. So one could speculate that in addition to the type of incentive (social vs. performance feedback), the nature of the incentive, i.e., whether it is of a more sustained (being observed for a period of time) or transient (feedback) nature, might influence which phase of performance monitoring is affected.

Flores et al. (2015) investigated young adults in a monetary incentive delay (MID) and a social incentive delay (SID) task while recording ERPs. In these tasks, an abstract incentive cue was shown, representing either no incentive, a monetary, or a social incentive that could be obtained for correct performance in the following trial. After having performed on the trial, monetary (blank coin, 10 or 20 cent coins) or social feedback (blank faces, faces with a slight or big smile) was presented. During incentive cue presentation, they found an enhanced attentional allocation (as reflected in the N1) and higher motivational salience (P2 and P3) to all cues signaling a potential reward. Additionally, there was a differential influence of the type of incentive: while social incentives affected processing in a very early time interval (larger N1), monetary incentives influenced later and more elaborate processing stages (larger P3). In the feedback processing phase, a larger FRN and P3 was found for all types of rewarding feedback. Moreover, monetary feedback resulted in a larger P2 and FRN than social feedback, reflecting a heightened motivational salience of monetary feedback (Flores et al., 2015). This is in line with a study by van den Berg et al. (2012), who found heightened motivational salience to monetary rewards as compared to abstract performance feedback indexed by a larger FRN and P3, and a study by Lin et al. (2012), who found faster learning in a monetary than a social reward condition in younger adults. Together, these studies clearly show that monetary and social reward feedback lead to enhanced performance monitoring and better learning and that younger adults seem to favor monetary over social rewards. Importantly, whether this greater sensitivity for monetary rewards is a specific effect found in younger adults and whether the same pattern of results would have been observed for potential losses is still an open question and beyond the scope of the reported studies.

Functional magnetic resonance imaging studies provide evidence that there are common as well as distinct brain areas involved in processing different types of rewards. Together, the existing data speak in favor of an anterior–posterior gradient in the orbitofrontal cortex, with primary reinforcers activating more posterior areas and more complex or abstract reinforcers activating more anterior regions (Izuma et al., 2008; Daniel and Pollmann, 2010; Lin et al., 2012; for meta-analysis, see Clithero and Rangel, 2014). However, the studies also show that there are shared brain structures responsible for the calculation of a subjective value, which allow to judge and compare different kinds of rewards or feedback on a common scale. These regions include the ventromedial PFC, posterior cingulate cortex, and striatum (Peters and Büchel, 2010; for a review, see Ruff and Fehr, 2014). To our knowledge, there are no similar fMRI or ERP studies comparing the processing of different types of rewards in older adults.

### Interim Conclusion

To conclude, the above studies demonstrate that motivational factors consistently enhance performance monitoring and improve performance in young adults. Neuroscientific studies examining age-related differences in this domain are rare and longitudinal data are missing completely, so it remains an open issue whether or under which circumstances motivational factors can enhance performance monitoring in older adults and diminish age-related impairments. Still, some preliminary conclusions can be drawn from the existing studies. First, adding additional information to the feedback stimulus seems to be an inappropriate way to examine motivational influences in older adults. This may be due to limited processing resources, which force older adults to focus on the most relevant properties of a feedback stimulus (cf. Herbert et al., 2011; Ferdinand and Kray, 2013). Thus, to examine whether reward magnitude influences monitoring processes in older adults, future studies need to find a way to either lower processing demands in general (e.g., by giving older adults more time to process feedback or by using very simple tasks) or to use feedback stimuli in which reward magnitude is incorporated in a more intuitive way (e.g., by using slightly vs. strongly smiling/frowning faces that can be processed holistically). Second, the existing studies indicate that under specific circumstances – when sufficient processing resources are available – positive incentives can ameliorate age-related performance deficits, while negative incentives can have deteriorating effects in older adults. However, this tentative conclusion is mainly based on the results of one behavioral study (Gorlick et al., 2013), which needs to be corroborated by convergent behavioral evidence from related paradigms and also by neuroscientific studies that can shed light on the cognitive mechanisms underlying this behavioral effect. Third, the studies reviewed above also hint at the idea that different types of incentives might be differentially motivating across the life span. While young adults seem highly susceptible to monetary rewards (Lin et al., 2012; van den Berg et al., 2012; Flores et al., 2015), social feedback seems especially effective in older adults (Gorlick et al., 2013). Nevertheless, studies comparing several kinds of incentives in both young and older adults and in the same paradigm are still missing.

## Motivational Influences on Cognitive Control

Cognitive control refers to the ability to regulate cognitive processes according to current task demands. This is particularly relevant when the task requires frequent updating of relevant information in working memory and protection against incoming irrelevant information (Miyake et al., 2000; Hofmann et al., 2012; Miyake and Friedman, 2012; Grange and Houghton, 2014). While performance monitoring allows us to identify situations in which additional control is necessary, the recruitment of additional task-specific cognitive resources is typically referred to as cognitive control *per se*. In line with the vulnerability of the PFC to aging, many empirical studies to date have identified pronounced age-associated deficits in tasks requiring cognitive control, specifically as task demands increase (for reviews, see Fabiani, 2012; Kray and Ferdinand, 2014). Hence, it is particularly relevant to examine whether motivational interventions could help to mitigate these age-related deficits in cognitive control.

The term *cognitive* control itself implies an inherent contrast to affective processing, raising the question whether and how motivation can influence purely cognitive mental operations. The precise nature of cognitive control is difficult to define, as it regulates the balance between many cognitive sub-processes necessary to successfully perform a task, the changing demands of the environment which may suddenly require immediate attention, and each individual’s overarching long-term goals such as the desire to perform well. In fact, enhanced control processes are often conceived as a filter blending out potentially distracting information and enabling us to focus exclusively on task-relevant details. However, the balance between long-term goals, the specific set of requirements of each task, and changing situational demands cannot be reached with a static filtering mechanism alone. Hence, in order to specify mechanisms of cognitive control, the environmental context and characteristics of the individual solving the task need to be taken into account. As participants vary in terms of task-relevant abilities, the amount of cognitive control that needs to be – and can be – mustered successfully is different for each participant and also between age groups. Moreover, many tasks can be approached successfully with more than one strategy, hence sometimes several types of qualitatively different cognitive control mechanisms can be feasible (see also Section “Potential Mechanisms of Motivation on Performance Monitoring and Cognitive Control in Old Age”). Finally, the recruitment of additional control processes is effortful and cannot be maintained for long periods of time, otherwise it would be optimal to keep cognitive control up-regulated for the duration of the task (Braver et al., 2007). More recent evidence suggests that participants differ in terms of how much effort they are willing to invest (Jimura et al., 2010) and choose the appropriate strategy maximizing not necessarily task performance, but rather a compromise between the maximum performance and effort invested. Hence, the regulation of cognitive control is not only influenced by task characteristics and intellectual abilities, but also by current motivational states (Chiew and Braver, 2011). Notably, strategies or types of cognitive control mechanisms used to approach a task can also differ as a function of age. For instance, older adults typically adopt a conservative response criterion emphasizing accuracy at the expense of speed regardless of task characteristics (e.g., Czernochowski et al., 2010). Hence, age may be an important factor determining which cognitive control mechanisms will be employed (Braver et al., 2007).

In the following, we will review empirical evidence on whether and how motivation can moderate the amount or type of cognitive control recruited in service of task performance (see **Table 2**). Note that some of the studies reported in this section examine performance monitoring, however, with a specific focus on how it may lead to enhanced cognitive control. In Section “Performance Feedback as Incentive,” we will examine how subsequent task performance and cognitive control are affected by performance feedback, which is often used in a non-systematic fashion in a general attempt to improve performance. Section “Processing of Reward Feedback” will summarize how experimentally introduced rewards or losses act as incentives for engaging in effortful cognitive control, whereas Section “Dissociating Specific Processing Stages With Incentive or Task-Preparation Cues” will focus on the role of different incentives cues and how they might differentially influence cognitive control.

**Table 2:** Behavioral and neuroscientific studies examining age differences in cognitive control.

Study	Method	Age groups	Motivational influence	Paradigm	Main results
Di Rosa et al., 2015	Behavioral	20–35 and 48–81 years (and Parkinson patients: 49–85 years)	Monetary rewards (+0.15€) for fast correct responses in a reward block and punishments (-0.15€) for slow and/or incorrect responses in a punishment block	Simon task	• For young adults, smaller Simon effects for blocks with potential losses than rewards • No systematic behavioral differences between reward and loss conditions for older adults • Young adults shifted to a more conservative response tendency in the loss condition, whereas older adults adopted a more conservative response criterion for the reward condition.
Drueke et al., 2012	Behavioral	23.9 and 70.5 years	Groups with block-wise performance feedback (mean RT) vs. without feedback	Flanker task	• In younger adults, performance feedback led to faster RTs and smaller RT congruency effects at the expense of increased error rates • In older adults, feedback led to higher error rates, but had no effect on RT
Drueke et al., 2015	fMRI	20–38 and 62–77 years	Trial-wise performance feedback (happy, sad or neutral smileys)	Flanker task	• Younger and older adults showed comparable reward-related activation • Positive feedback elicited the strongest striatal and amygdala activation and slightly faster reaction times in older and younger adults
Schmitt et al., 2015	ERPs	19–28 and 69–78 years	Monetary incentives (gains, losses, or neutral)	Modified AX-CPT	• Age-invariant enhanced processing of gain and loss as compared to neutral cues • Younger adults were particularly susceptible to potential losses as indexed by improved context maintenance (larger CNV) prior to the probe and increased conflict detection (N450) and resolution (sustained positivity) during response selection after loss cues • Older adults showed enhanced cognitive control during task preparation (larger cue-locked P3b) and during response preparation and execution (prolonged probe-locked P3b) after gain and loss cues
Schmitt et al., 2017	ERPs	65–76 and 69–78 years	Monetary incentives (gains, losses, or neutral)	Modified AX-CPT	• When incentives are presented in a block-wise manner, older adults initially process cues signaling potential losses more strongly, but invest more cognitive resources in preparatory processes like context updating in conditions with potential gains
Spaniol et al., 2015	fMRI	20–33 and 60–78 years	Monetary incentives (Win $5, Win $0, Lose $5, and Lose $0)	Monetary incentive delay (MID) task	• Two significant latent variables representing distinct incentive-related activation patterns: (1) Robust activation of the reward network, not modulated by age (2) Peaking 10 s after cue onset, reduced deactivation of default-network regions and increased activation of prefrontal cognitive-control regions in older adults
Wild-Wall et al., 2009	ERPs	23.7 and 57.5 years	Verbal performance feedback (correct/incorrect)	Motor timing task	• Both age groups more accurate following positive compared to negative feedback • Only young adults improved following negative performance feedback, older adults more likely to commit another error following an error feedback • Only for young adults, differences in FRN amplitudes between error and correct feedback correspond to the percentage of correct responses • Reduced differentiation of FRN amplitudes between correct and incorrect trials for older adults
Williams et al., 2017	Behavioral	18–34 and 60–82 years	Gains and losses	Attention network test (ANT)	• Both types of rewards improved overall performance (dividing RT by accuracy) across groups, but both effects were more pronounced for younger adults • Rewards not only reduced overall RTs, but also considerably increased interference effects in terms of accuracy (modified speed/accuracy tradeoff rather than enhanced cognitive control)

### Performance Feedback as Incentive

One way to motivationally influence performance is to give general performance feedback. Often a summary of performance in the previous block is provided before participants can take a brief pause from the task and have the opportunity to reflect on it. In fact, many studies in which motivation is not studied experimentally rely on such an informal way to remind participants that there is room for improvement, sometimes by providing other participants’ outcome as a social reference or by emphasizing either response speed or accuracy. However, this type of feedback does not always lead to the expected improvements in performance, but can also have detrimental effects, especially for older adults. For instance, Drueke et al. (2012) provided block-wise feedback to young and older participants. Performance feedback was given to half of the participants in each age group, based on response times during a flanker task. For the young, feedback led to faster reaction times (RT) at the expense of reduced accuracy. Notably, the feedback-induced focus on response speed also affected control processes: RT congruency effects (i.e., the difference between incongruent and congruent trials) were reduced for the feedback group compared to their peers who did not receive performance feedback, suggesting enhanced processing of relevant stimuli and/or more successful inhibition of irrelevant stimuli. By contrast, older adults’ performance speed was not modulated by performance feedback, but the feedback group of older adults still committed more errors (Drueke et al., 2012), suggesting that older adults failed to modulate their task approach successfully. Although this initial study exclusively relied on behavioral data, one could speculate that the older adults in the feedback group misallocated attentional resources otherwise available for successful task performance. However, it remains unclear how cognitive control mechanisms are modulated by this block-wise intervention resuming the performance level of many trials.

In general, performance feedback should be most informative for future response selection when given on a trial-by-trial basis. According to the conflict monitoring account (Botvinick et al., 2001), negative performance feedback in particular provides potentially useful information, signaling the need to revise the current response strategy and to recruit additional cognitive control processes. However, even negative performance feedback given after each response does not necessarily promote cognitive control processes. For instance, in an fMRI version of their flanker task, Drueke et al. (2015) provided performance feedback to young and older participants on a trial-by-trial basis. In addition, performance remained unevaluated in one third of all trials. For both age groups, subsequent RTs were faster after positive compared to neutral feedback, whereas negative feedback did not modulate performance. Imaging data revealed that task-relevant areas and those related to reward processing were active to a similar extent in both age groups, whereas older adults also activated additional brain regions (Drueke et al., 2015). This pattern of unspecific over-activation or de-differentiation of brain activity is often observed in older adults, and usually taken as evidence for an – not necessarily successful – attempt to compensate age-related deficits (for a review, see Reuter-Lorenz and Lustig, 2005). Similarly, in an ERP study, young and older adults were asked to produce precisely timed motor responses and received verbal feedback (correct/incorrect) after each response (Wild-Wall et al., 2009). Only young adults successfully implemented negative performance feedback to promote subsequent performance, whereas older adults were much more likely to commit another error following an error feedback (conditional probability *p* = 0.67) compared to following a correct response feedback (*p* = 0.33). The authors attributed this particular difficulty in regulating response speed to age-associated deficits in the dopamine system responsible for precise motor timing. In line with the results reported above, both age groups were more accurate in producing a precisely timed motor response following positive compared to negative feedback (Wild-Wall et al., 2009). Electrophysiological data collected during this task can provide useful information on the mechanisms underlying feedback processing and potential cognitive control processes. For young adults, differences in FRN amplitudes between error and correct feedback corresponded to the percentage of correct responses, suggesting that feedback information was used to adjust the timing of subsequent responses, although negative feedback did not contain information on whether the response was given too slow or too fast. Notably, no such association was observed in older adults, who also showed a reduced differentiation of FRN amplitudes between correct and incorrect trials, implying a high level of conflict across conditions. These results suggest that older adults had difficulties to differentiate conditions in which enhanced cognitive control was necessary (Wild-Wall et al., 2009).

Notably, in the studies reviewed above, the presence or absence of cognitive control mechanisms is inferred indirectly by analyzing how performance in subsequent trials is modulated as a consequence of negative or positive external feedback regarding response speed. Together, these findings imply that only younger adults successfully re-vise their current task approach and manage to improve their performance in subsequent trials. It remains unclear why older adults do not succeed in improving their performance following negative feedback: In line with the evidence reviewed in Section “Motivational Influences on Performance Monitoring,” electrophysiological data suggest that feedback processing as a signal to initiate cognitive control processes remains relatively intact in old age, as long as task demands are not too high. However, older adults often show heightened conflict processing even for correct trials (cf. Nessler et al., 2007; Czernochowski et al., 2010). In line with these less differentiated conflict signals, older adults may have difficulty to resolve the experienced conflict by recruiting additional control processes, in particular when response conflict is accumulating over several trials. Moreover, attempts to recruit additional control processes may be less successful in older adults.

In addition, intrinsic and long-term motivational age differences participants bring to the lab are likely to affect how performance feedback is being processed and whether or not participants will be willing and able to recruit additional cognitive control processes. Positive feedback – signaling that the current task approach is suitable and should be continued, potentially with enhanced effort – may be more instrumental in optimizing task performance than negative feedback and has similar consequences for young and older adults. Conversely, negative performance feedback can tie attentional resources and distract participants in subsequent trials, specifically individuals who are highly motivated to perform well but do not have much room for improvement. For instance, when feedback is based predominantly on speed of processing, older adults may find themselves unable to respond faster. In this scenario, negative performance feedback is presumably also particularly salient for older adults who like to be re-assured that their performance level is still age-appropriate (cf. Baltes and Baltes, 1990; Ebner et al., 2006; Brandtstädter, 2009). Hence, older adults can be particularly sensitive to negative feedback, which can have negative rather than beneficial effects. As a result, in the studies reviewed above, only young adults were sometimes able to successfully recruit cognitive control following negative performance feedback.

### Processing of Reward Feedback

A more direct way to examine the influence of motivation is to provide rewards for (rapid and) correct task performance, often consistently across many trials (i.e., block-wise modulation). While this approach will not capture potential trial-by-trial fluctuations of cognitive control, some strategic adjustments may rather be reflected in tonic activity. Taking advantage of this approach, Locke and Braver (2008) were the first to compare young adults performing the AX-CPT task in blocks associated with either monetary rewards or losses compared to baseline performance. In this paradigm, participants are asked to respond to a target X whenever it is preceded by a specific cue A and to withhold a response in all other cue-target combinations (AY, BX, and BY and sometimes also No-Go trials, which occur in around 10% of trials each). In line with enhanced advance preparation, young participants were considerably faster to respond on the frequent AX trials during the reward block compared to either baseline or monetary loss condition. However, this condition was also associated with a selective increase in AY errors, suggesting that faster RTs on the majority of trials were achieved at the expense of increased errors in this rare trial type. Imaging data identified sustained activity in a network of right-lateralized regions including lateral PFC, right parietal and dorsal medio-frontal cortex, presumably reflecting context maintenance underlying this behavioral reward effect. Conversely, for the monetary loss condition, AX errors increased despite slow RTs, but NoGo errors were substantially reduced, suggesting that participants adopted a more cautious response criterion (Locke and Braver, 2008; see also Chiew and Braver, 2014). Notably, increased reliance on advance preparation is only observed when rewards are provided contingent on correct performance, whereas performance was not modulated for a group of young adults who received rewards randomly as *a gift* and hence unrelated to performance (Fröber and Dreisbach, 2016, see also Fröber and Dreisbach, 2014). Thus, enhanced cognitive control is only recruited in service of optimizing performance when superior performance is instrumental to gain rewards.

To summarize, rewards consistently modulate young adults’ behavior when provided contingent on individual performance. Positive incentives promote fast responses presumably via advance preparation, albeit sometimes at the expense of increased errors rates in rare task conditions requiring response inhibition. In these reward conditions, neuroimaging studies have identified a network of sustained activity predominantly in lateral prefrontal brain regions consistent with task maintenance. Introducing penalty incentives does not activate these areas and tends to have weaker impact on performance, associated with slower responses and a more conservative response criterion.

To our knowledge, only three studies so far explicitly studied rewards in the context of aging in cognitive control paradigms. Di Rosa et al. (2015) compared sequential blocks of monetary rewards or losses (0.15€) between young and older participants. During a Simon paradigm, a bonus was awarded for fast and correct responses, whereas slow or incorrect responses were associated with losses. For young adults, Simon effects (i.e., differences in accuracy for incongruent vs. congruent trials) were smaller for blocks with potential losses than rewards, suggesting more efficient processing for the loss condition. For older adults, behavioral differences between reward and loss conditions were less systematic. In addition, young adults shifted to a more conservative response tendency in the loss condition, whereas older adults adopted did so in the reward condition. These opposing patterns imply that rewards and losses had a qualitatively different impact in each age group (Di Rosa et al., 2015). In a task combining flanker and attentional cueing, Williams et al. (2017) provided incentives on randomly intermixed trials. Young and older participants could either gain or avoid loosing $.10 from a pre-experimental balance of $30 (between-participant comparisons). A combined performance index was used to compare the age groups, indicating that both types of rewards improved overall performance across groups, but both effects were more pronounced for younger adults (Williams et al., 2017). However, as increasingly fast and accurate responses were rewarded/not penalized in this paradigm, rewards not only reduced overall RTs, but also considerably increased interference effects in terms of accuracy. This pattern of results suggests that incentives encouraged participants to prioritize speed at the expense of overall accuracy rather than to enhance cognitive control. Using functional imaging, Spaniol et al. (2015) compared how young and older adults evaluate monetary incentive cues ($5 vs. $0) signaling how much money could be gained or lost in the subsequent trial. Performance in a target detection task was equated between age groups by adjusting the target duration based on each individual’s performance in the previous trial. Still, cumulative earnings were higher for young adults, who modulated their performance as a function of incentives much more than did older adults. Conversely, associated fMRI data revealed age-invariant activity in the reward circuitry. However, in line with the behavioral earnings, only young adults de-activated the default network for the gain/loss condition more than for the $0 condition. Despite the age-invariant reward-activation, the reverse pattern (i.e., increased task preparation for the non-incentive conditions) was observed in older adults, suggesting difficulties in modulating preparation as a function of changing task incentives (Spaniol et al., 2015).

To summarize, tentative evidence available so far suggests that behavioral age differences in cognitive control persist when monetary rewards or losses are added to the informational content of performance feedback. Older adults appear to scarcely modify their behavior based on rewards. Conversely, young adults incorporate reward contingencies more flexibly, in particular when emphasis is placed on response speed. Hence, young adults might revise their current strategy, but not necessarily recruit additional control processes successfully (e.g., when a focus on response speed is associated with reduced accuracy). As a result, the precise impact of incentives often depends on which aspect of behavioral performance is examined. Notably, the scarce extant data point to an incentive-based shift in task strategy for the young, but not in older adults. Imaging data complement this picture with unspecific or de-differentiated brain activation patterns during advance preparation (cf. Czernochowski, 2011). However, the precise mechanisms how motivation can modulate cognitive control and whether age mediates these reward effects remain unclear.

### Dissociating Specific Processing Stages With Incentive or Task-Preparation Cues

Instead of providing rewarding feedback in randomized trials or blocks of trials, incentives can also be associated with selective aspects of a complex task. For instance, when one of two tasks during a task-switching paradigm is consistently associated with a bonus, RTs in young adults decrease for the bonus task, and specifically for trials requiring enhanced control due to a switch between tasks. Remarkably, performance for the bonus task also improved for the majority of trials in which no bonus was delivered, and overall task performance improved as an unpredicted side effect, implying that enhanced motivation for one task may not be easy to modulate in a transient manner (Kleinsorge and Rinkenauer, 2012, Exp 1, cf. Section 4.2). Krebs et al. (2013) showed similar effects for young adults in a Stroop task with a fixed association between two out of four colors and potential monetary gains or losses ($.10). Moreover, they found that rewards modulated early fronto-central and occipital ERP components (like the N200 and P300), in line with enhanced attentional processing of reward-related target information and enhanced behavioral performance. Also, conflict-related ERP components (like the N_inc_ and LPC) were observed considerably earlier, suggesting that reward prospects modulate the temporal dynamics of conflict processing (Krebs et al., 2013). Thus, a fixed association between rewarded tasks or stimuli results in enhanced processing for reward-related stimuli, presumably affecting early attentional stages of stimulus evaluation as well as conflict-processing. However, no data on older adults and potential age differences in selectively rewarding certain task aspects are currently available.

Different processing stages can also be dissociated using high-resolution pupillometry. For instance, Chiew and Braver (2013) provided incentives during the AX-CPT on a trial-by-trial basis in a potential reward block as compared to a block without incentives to young participants. Pupil dilation effects were observed prior to the probe during context cue maintenance, along with more efficient performance on AX trials and an increased error rate on AY trials, suggesting that participants considerably relied on advance preparation. In addition to these transient effects, sustained pupil dilation effects were observed during the entire potential reward block, indicating that reward incentives increased the use of cognitive control on a trial-wise as well as block-wise fashion (Chiew and Braver, 2013).

In the vast majority of studies reviewed so far, participants were provided with cues signaling an incentive for the upcoming trial, allowing for task-unspecific advance preparation. Another approach is to provide participants with advance information about the upcoming task, enabling them to prepare more specifically. For instance, Chiew and Braver (2016) compared the effects of reward cues with cues indicating whether the upcoming flanker trial would consist of congruent, neutral, or incongruent stimuli and used drops of apple juice as primary reinforcer in thirsty young participants. Only when reward cues were associated with task-informative cues, cognitive control was enhanced as evident in reduced interference costs.

To the best of our knowledge, only two studies to date examined how incentives specifically modulate temporally distinct cognitive control processes in older adults. In a study by Schmitt et al. (2015), young and older participants performed a version of the AX-CPT including the trial-wise presentation of incentive cues announcing potential monetary gains, losses, or neutral outcomes depending on performance. ERPs revealed enhanced processing of gain and loss compared to neutral cues that was age-invariant. Additionally, younger adults were particularly susceptible to potential losses as indexed by improved preparatory context maintenance (larger CNV) prior to probe presentation as well as increased conflict detection (N450) and resolution (sustained positivity) during response selection whenever incorrect responding would have led to a loss. Conversely, older adults showed enhanced control processes during task preparation (larger cue-locked P3b) and during response preparation and execution (prolonged probe-locked P3b) after gain and loss cues (Schmitt et al., 2015). In a follow-up study, Schmitt et al. (2017) demonstrated that a trial-wise presentation of incentive cues in older adults results in different effects than a block-wise presentation, presumably due to stronger demands on processing resources. When incentives were presented in a block-wise manner, older adults initially processed cues signaling potential losses more strongly, but later during the AX-CPT invested more cognitive resources in preparatory processes like context updating in conditions with potential gains. Hence, how positive and negative incentive cues influence cognitive control in older adults depends on the demands of cue processing.

### Interim Conclusion

In a more or less systematic attempt to modulate cognitive control, various ways of providing performance feedback have been used, and some of the reported inconsistencies can be attributed to subtle differences in the type or timing of this feedback. Two conclusions can be derived from these studies: performance feedback has larger impacts on young than older adults, and positive feedback improves performance more consistently than negative feedback. Conversely, the extent to which negative feedback is instrumental in improving performance depends on the exact paradigm and available processing resources, hence older adults’ performance may suffer rather than benefit from performance feedback. Cognitive accounts have tried to explain this phenomenon with unsuccessful attempts to up-regulate control processes and less differentiated conflict signals accumulating over several trials. Alternatively, age differences in long-time motivations like the desire to perform well or anxiety related to age-related performance decrements could be responsible for the failure to benefit from negative performance feedback, which would both render negative performance feedback particular salient for older adults. As a result, attentional resources would be taken away from pursuing the task at hand. The scarce available evidence suggests that adding monetary rewards or losses to informational performance feedback does not change this general pattern of results. Indeed, behavioral age differences in cognitive control might even increase as young adults incorporate reward contingencies more flexibly and might enhance cognitive control based on reward prospect, whereas older adults barely modify their behavior based on motivational incentives (Williams et al., 2017), or change their response pattern with respect to speed-accuracy tradeoffs to the opposite direction compared to young adults (Di Rosa et al., 2015).

Critically, cognitive control mechanisms are typically inferred indirectly by analyzing how performance in subsequent trials is modulated as a consequence of feedback. In order to determine how differential control processes may be targeted by incentives, more specific task manipulations are necessary. One promising way is to provide advance cues to specifically assess the effects of incentives or to allow for task-specific preparation. During incentive cue processing, the extant data consistently point to an incentive-based shift in task strategy consistent with enhanced preparation for reward-trials in the young. With respect to aging, evidence is scarce: only one fMRI study examined reward cue processing in older adults. Despite age-invariant activity in the reward circuitry, older adults increased task preparation for the non-incentive conditions and thus earned fewer rewards compared to the young who showed the opposite pattern (Spaniol et al., 2015). Similarly, two recent ERP studies provide evidence that older adults modulate early preparatory cognitive processes and late response-selection stages based on reward or loss incentive cues, however, rely on different processes compared to young adults (Schmitt et al., 2015, 2017). Notably, timing is critical for efficient use of both incentive and task-specific cues, and enhanced cognitive control is only observed when sufficient time is available to process incentive cues and hence allow advance preparation (Chiew and Braver, 2016). Notably, older adults often require additional time to prepare for an upcoming task, and age differences in recruiting cognitive control are smaller for long as compared to shorter preparatory intervals (cf. Czernochowski, 2011).

Taken together, age differences are often accentuated rather than diminished when providing performance feedback or monetary rewards in an attempt to enhance motivation. Only under specific circumstances, control processes appear to be effectively enhanced by motivational interventions in older adults, for instance when positive feedback is provided based on performance aspects with sufficient room to improve, or when early advance cues provide the opportunity to make up for less efficient processing in old age. However, the precise mechanisms underlying successful motivational interventions currently remain open.

## Potential Mechanisms of Motivation on Performance Monitoring and Cognitive Control in Old Age

In order to fully understand cognitive processing in old age, it is critical to take into account neurobiological factors associated with aging and potentially underlying observed changes in cognition. Despite an immense variability of cognitive functions observed in old age (Fabiani, 2012) and the potential role of compensation and functional re-organization to mitigate existing neurological deficits (e.g., Stern, 2002; Reuter-Lorenz and Lustig, 2005; Czernochowski et al., 2008), two neurobiological factors have been consistently implicated in cognitive aging: in terms of brain structures, negative consequences of aging are particularly pronounced in the PFC (for reviews, see Raz, 2000; Samson and Barnes, 2013), and specifically the dorsolateral PFC (e.g., MacPherson et al., 2002), implying that at least some deficits in cognitive control operations relying on these brain areas are characteristic for older individuals. In terms of neurotransmitter systems, aging affects predominantly dopaminergic pathways, also responsible for motivation and reward processing (for reviews, see Bäckman et al., 2000). In line with the pivotal role of dopamine for reward processing, pharmacological increases in dopamine levels further enhance the differentiation between rewarded and non-rewarded conditions in young adults relative to placebo (e.g., Weis et al., 2012), implying that reduced levels of dopamine might directly underlie at least some of the observed age differences reviewed above. However, large inter-individual variability and distinct optimal dopamine levels depending on the precise nature of each cognitive control task complicate the study of the precise dose-dependent effects of dopamine (Cools and D’Esposito, 2011).

One very influential neurobiological model of aging and its impact on cognitive control that might be able to incorporate the influence of motivation on cognition proposes that disruptions in dopaminergic neurotransmission can be understood as a common underlying mechanism for a variety of age-associated deficits across multiple cognitive domains (Braver and Barch, 2002). According to this model, the dorsolateral PFC and dopaminergic projections to this region are held to serve three distinct functions: (1) active maintenance of context representations in working memory, (2) biasing of local representations to prioritize currently relevant information according to this context, and (3) a gating mechanism subserved by phasic releases of dopamine to allow newly relevant information to be used for updating context information when appropriate. Dopaminergic projections to the dorsolateral PFC are believed to regulate the balance between stable context representations, necessary to inhibit currently irrelevant information in the pursuit of goal-directed behavior, and the flexibility to update context information according to new demands or task instructions. Of particular relevance for the question of how motivation might affect cognition, the phasic release of dopamine may trigger the updating of context information by signaling reward-predictive information to be represented as context. When these dopaminergic pathways are disturbed in the aging process, older adults will experience difficulty in the active representation of context in working memory, causing cognitive deficits across various cognitive domains. Notably, context updating associated with the phasic release of dopamine appears most vulnerable to aging, and deficits in this particular aspect of cognitive functioning are observed already relatively early in the aging process. Conversely, the tonic release of dopamine has been implicated in the active maintenance of context information and seems susceptible to more advanced age only. Ultimately, these changes negatively impact various aspects of cognitive control, which relies on an active representation of current task rules and goals (Braver and Barch, 2002). Hence, it is of utmost importance to know how these age deficits can be ameliorated. The above literature review demonstrated that using rewards to influence performance monitoring and cognitive control might be a promising way to do this. However, empirical research to date has only begun to elucidate the precise circumstances in which enhanced motivation will have the desired effects.

### The Dual Mechanisms of Control (DMC) Framework

As mentioned above, the conflict monitoring account (Botvinick et al., 2001) proposes that the detection of response conflict, provided externally by negative performance feedback or internally by realizing that we just committed an error, can trigger the recruitment of additional control resources to promote future task performance. This mechanism can prevent further response conflict in subsequent trials until control resources are no longer deemed necessary and hence are down-regulated again. Extending this model by introducing an alternative to this so-called *reactive control*, the DMC framework (Braver et al., 2007; Braver, 2012) posits that additional control processes can be activated proactively when upcoming response conflict can be expected, for instance when participants are required to maintain a task context during sustained attention tasks. Hence, *proactive control* is a more stable and temporally sustained process resulting in both fast and accurate responses, as conflict does not need to be detected and resolved. As proactive control in particular is not feasible in situations of unexpected response conflict and requires considerable attentional resources to be maintained over time, participants switch to a more reactive control-mode when necessary. The reactive control process, in turn, is subject to considerable trial-by-trial variability, and particularly useful in a self-paced task when responses can be withheld as response conflict builds up due to simultaneous activation of response tendencies until the appropriate response selection can be employed to allow for slow, but accurate responding. Hence, both control processes can be used flexibly according to task demands and attentional resources and are subject to continuous fluctuations. Therefore, the DMC framework has important implications for the role of motivation in moderating cognitive control. In recent years, empirical evidence for the DMC has dissociated proactive vs. reactive control modes and its putative underlying neuronal substrates using fMRI (e.g., Locke and Braver, 2008), ERPs (e.g., Czernochowski, 2014; Arbula et al., 2016) or high-resolution pupillometry (Chiew and Braver, 2013). However, it is often not sufficient to allow for advance preparation when trying to elicit proactive control, even when there is room for improvement in individual task performance. Notably, prior work has consistently demonstrated that young adults are particularly likely to activate the demanding proactive control mode promoting advanced preparation when motivation is enhanced via performance-contingent reward incentives.

In the context of aging research, the DMC model is particularly intriguing as older individuals have specific deficits in context processing as a result of neurodegenerative decline in the prefrontal dopamine system (cf. Braver et al., 2001), as detailed above. By comparing fMRI activity during the AX-CPT paradigm, Paxton et al. (2008) provided empirical support for a dissociable time course of control processes in young and older participants when no incentives or external performance feedback were provided. In line with a proactive control strategy and the results reviewed above, right dorsolateral PFC activity was observed during the cue-target interval for young adults. By contrast, older adults only activated this area after target onset, suggesting a shift to a reactive control strategy (Paxton et al., 2008). This initial finding and the specific time course proposed for control processes in the DMC, provide a useful tool to examine reactive and proactive control processes in young and older adults. For instance, to identify an ERP correlate for reactive control, Czernochowski et al. (2010) compared response-locked ERP averages during a cued task-switch paradigm, in which conflict increased along with task difficulty for older adults in particular. Slow and fast responses for each task condition were selected to contrast responses based predominantly on reactive or proactive control. For young adults, a negative ERP modulation preceding the response at left frontal electrode sites (pre-response negativity or PRN) was observed along with behavioral costs selectively for slow responses during high-conflict trials taken to reflect predominantly reactive control. By contrast, as predicted by the DMC, the corresponding ERP modulation as well as pronounced RT costs were observed less selectively in older adults, and related to slow, but very accurate responses. Together, these results suggest that young adults employed reactive control only when necessary to support performance for high conflict trials, whereas older adults relied predominantly on reactive control, as evident in additional neuronal activity preceding the response.

The DMC also has important implications regarding the role of motivation to mitigate age differences in cognitive control. Notably, the DMC model explicitly posits that older adults suffer from a specific neurocognitive deficit in maintaining context representations. Hence, older adults would be compelled to rely predominantly on reactive control processes. In line with a true deficit in recruiting proactive control, age differences in terms of qualitatively different modes of controlled processing should be relatively stable across time and experimental incentive conditions. Alternatively, reactive control – sharing a number of characteristics with prioritizing accuracy over speed of responding as typically observed in older adults (cf. Rabbitt, 1979) – might at least to some extent reflect a deliberate or customary choice of strategy. In this scenario, older adults’ preference for reactive control should be less stable and modifiable by introducing incentives. As reviewed in Section “Motivational Influences on Cognitive Control,” introducing monetary rewards or penalties does not appear sufficient to induce qualitative changes in the type of control processes recruited by older adults. By contrast to young adults, a lack of motivation to select more effortful strategies to promote task performance does not appear to be the critical limiting factor for older adults (see also Braver et al., 2014). In fact, one of the first studies to report changes in control modes in older adults relied on a strategy training rather than reward incentives (Braver et al., 2009). This finding has important implications: despite age-related neurocognitive changes in the dopaminergic system and structural changes to dorsolateral PFC, the use of proactive control is feasible in older adults. However, at the same time this finding underlines the fact that old age is associated with a less flexible use of cognitive control. For young adults, providing monetary incentives or strategy training promotes proactive control, whereas potential monetary losses or introducing NoGo-trials discourages the use of proactive control (Gonthier et al., 2016). Monetary incentives or performance feedback may promote modes of cognitive control in older adults only under specific circumstances which are currently not evaluated in sufficient detail, for instance when sufficient time and processing resources are available (e.g., Schmitt et al., 2017). Another step may include more explicit explanations on the relative importance of rapid and correct responses for many laboratory paradigms, which typically differ between older and young adults.

## Open Issues, Caveats, and Future Directions

On a more general note, motivational factors are not only a promising tool for mitigating age-related reductions in performance monitoring and cognitive control, intrinsic motivational age differences participants bring to the lab are likely to affect how incentives are processed and which ones are prioritized. In line with this, older participants often appear particularly motivated to reach good performance, although this is rarely formally assessed (for an exception, see Staub et al., 2014). To further complicate matters, increased motivation is likely to result in increased effort mobilized for the task, but this may not necessarily translate to better performance, particularly in old age. For instance, Ennis et al. (2013) compared young and older participants during a modified Sternberg memory search task and compared how much effort each group invested, as indexed by an increase in systolic blood pressure. Importantly, they also varied objective task difficulty and recorded motivation to do well as well as perceived control. In line with prior investigations, memory performance declined as a function of task difficulty and age. Extending prior work and in contrast to behavioral performance levels, findings suggest that older compared to younger participants invested more effort at all levels of task difficulty. However, as time on task increased and at the highest level of difficulty, older adults disengaged from the task and reduced their effort more than the young. Notably, motivation to do well and higher perceived control was associated with increased effort only for older participants (Ennis et al., 2013). Thus, not only individual participants but also young and older participants vary systematically in terms of how much effort they are willing – and able – to allocate to a task at hand. Unfortunately, increased effort does not translate linearly to better performance and also depends on task difficulty, time on task, and motivation to perform well. Finally, participants in aging research vary tremendously in terms of chronological age (in the studies reviewed above, age range extends from 48 to 85 years), but also other factors not routinely reported (years and type of education, health parameters, etc.). As a result, even before introducing experimental manipulations, we might compare a highly selective group of older adults in full sprint with young adults routinely jogging at a leisurely pace. In such a scenario, it should not be too surprising that only young adult samples can be motivated by incentives to improve/modulate performance. Given the increased variability in aging, it is likely that sub-groups may use cognitive reserve to compensate difficulties (cf. Czernochowski et al., 2008). As long as older adults have room to compensate by recruiting additional brain areas, there will be no general behavioral age differences in performance levels unless more sophisticated methods are used to quantify evidence for different strategies. Neuro-scientific studies have begun to help to dissociate behavioral differences and to which extent they might be a result of extensive efforts to compensate age-related decline.

A second open issue concerns the nature and complexity of paradigms used in aging research. Overall difficulty, type of control needed to perform successfully, and precise timing vary considerably, and can account for at least some of the inconsistencies reported above. As reviewed above, timing and the role of speeded responses may be critical for advance preparation, but also has important implications for performance feedback, in particular when older adults invariably receive negative performance feedback based on their overall slower responses and relatively strict algorithms defining response deadlines. To avoid inconclusive results in future studies, some means of comparison of older adult samples across studies would be extremely useful. For instance, including basic measures of working memory spans or performance in simple speed tasks without major demands on cognitive control could help to create a more meaningful overview of performance in old age. Conversely, paradigms used for young and older adults sometimes may need to vary in important details. For instance, the timing of advance cues is of particular relevance when comparing age groups, as older adults typically need additional time for task preparation but may overcome their difficulties with longer cue-target intervals (Czernochowski, 2011).

Particularly useful to delineate specific age-related differences are task variants demonstrating the lack of age differences for selected conditions, or even paradox performance advantages (e.g., weak context representations in the AX-CPT reducing interference in B-X trials). Moreover, indices of effort put into task by each individual and age group, independent of behavioral outcome, could help to shed more light on complex motivation–cognition interactions and avoid circular argumentations. The precise implications of a lack of controlled processing in young adults with healthy PFC functions for potential aging effects in the same task remain unclear. Again, neuro-scientific measures can be fruitful for this endeavor in the future, but at present inter-individual variability in the strategies employed during a specific task or in cognitive reserve more generally, limit viable interpretations of task-unspecific brain activations. The DMC framework has been very instrumental in dissociating cognitive control processes, and spurred empirical evidence of how motivational factors can moderate cognitive control in young and older adults. Conversely, a clear taxonomy of paradigms used to investigate these effects and their implications cognitive control and its specific timing (e.g., with-in trial for self-paced designs or between-trial effects for speeded designs) is still missing to date.

## Conclusion

We report empirical findings demonstrating that incentives can modulate performance monitoring and cognitive control. Although studies examining age-related differences across adulthood are scarce, the existing ones indicate that under specific circumstances incentives can ameliorate age-related deficits. However, even apparently subtle inconsistencies between paradigms may have important implications seriously limiting the conclusions that can be drawn at this point. Clearly, future research is needed to clarify (a) the specific circumstances under which incentives can reduce rather than enlarge age-related impairments, (b) which types of incentives are most effective in which age group, and (c) the role of increased effort in overcoming individual differences in ability underlying performance differences in young but especially among older adults. Critically, due to compensatory use of differential strategies or increased effort, the true amount of age-related deficits may be systematically underestimated. While both ways to ameliorate performance deficits are useful and should be encouraged, they cannot be maintained over a longer period, hence it is important to differentiate between typical performance in old age and what can be achieved using all available resources and highest level of motivation. To achieve this, careful and innovative analyses on several aspects of behavioral performance are needed along with neuroscientific methods that together can shed light on the specific neuronal substrates and cognitive processes involved in successful task performance. We also noted several general caveats impeding research in this area, including the fact that participants differ in their long-term motivation to take part in research which can mask age-differences as long as (some) older adults are able to compensate. This substantiates the need to focus on neural activation patterns that can uncover both cognitive and motivational mechanisms underlying age differences in performance.

## Author Contributions

All authors listed have made a substantial, direct and intellectual contribution to the work, and approved it for publication.

## Conflict of Interest Statement

The authors declare that the research was conducted in the absence of any commercial or financial relationships that could be construed as a potential conflict of interest.
